# Characterization of a filamentous biofilm community established in a cellulose-fed microbial fuel cell

**DOI:** 10.1186/1471-2180-8-6

**Published:** 2008-01-10

**Authors:** Shun'ichi Ishii, Takefumi Shimoyama, Yasuaki Hotta, Kazuya Watanabe

**Affiliations:** 1 Marine Biotechnology Institute, Heita, Kamaishi, Iwate 026-000, Japan; 2 Central Research Institute of Oral Science, School of Dentistry, Asahi University, Hozumi, Mizuho, Gifu 501-0296, Japan; 3 Institute for Biological Resources and Function, AIST, Higashi, Tsukuba, Ibaraki 305-8566, Japan

## Abstract

**Background:**

Microbial fuel cells (MFCs) are devices that exploit microorganisms to generate electric power from organic matter. Despite the development of efficient MFC reactors, the microbiology of electricity generation remains to be sufficiently understood.

**Results:**

A laboratory-scale two-chamber microbial fuel cell (MFC) was inoculated with rice paddy field soil and fed cellulose as the carbon and energy source. Electricity-generating microorganisms were enriched by subculturing biofilms that attached onto anode electrodes. An electric current of 0.2 mA was generated from the first enrichment culture, and ratios of the major metabolites (e.g., electric current, methane and acetate) became stable after the forth enrichment. In order to investigate the electrogenic microbial community in the anode biofilm, it was morphologically analyzed by electron microscopy, and community members were phylogenetically identified by 16S rRNA gene clone-library analyses. Electron microscopy revealed that filamentous cells and rod-shaped cells with prosthecae-like filamentous appendages were abundantly present in the biofilm. Filamentous cells and appendages were interconnected via thin filaments. The clone library analyses frequently detected phylotypes affiliated with *Clostridiales*, *Chloroflexi*, *Rhizobiales *and *Methanobacterium*. Fluorescence in-situ hybridization revealed that the *Rhizobiales *population represented rod-shaped cells with filamentous appendages and constituted over 30% of the total population.

**Conclusion:**

Bacteria affiliated with the *Rhizobiales *constituted the major population in the cellulose-fed MFC and exhibited unique morphology with filamentous appendages. They are considered to play important roles in the cellulose-degrading electrogenic community.

## Background

Microbial fuel cells (MFCs) are devices that exploit microorganisms to generate electric power from organic matters and potentially applicable to wastewater treatment and energy recovery from organic wastes [1-4]. Recent technical developments of MFC processes are noteworthy, and the power output of MFC has been rapidly increasing in recent several years [1]. Some workers consider that MFCs will be practically applied in the near future, but further studies, such as stability improvements and scale-up evaluations, are necessary for that.

A conventional MFC reactor is comprised of two chambers, the anode and cathode chambers [1-4]. In the anode chamber, organic matter is oxidized to carbon dioxide by microorganisms under anaerobic conditions, reducing equivalents are discharged to the anode as electrons, and these electrons are transferred to the cathode. Protons are simultaneously generated in the anode chamber, passively transferred to the cathode chamber through a membrane and react with oxygen molecules on the cathode electrode to form water molecules. All these steps influence the total efficiency (e.g., power density, coulombic efficiency and organic-loading rate [1]) of a MFC process, and each of these steps has been a subject for technical improvement. For example, the membrane has been optimized to achieve efficient proton transfer [5] and the cathode was modified to accelerate the proton-reducing reaction [6].

It has been suggested that in addition to process optimization, the understanding of ecology and physiology of electricity-generating microorganisms (EGMs in this study; other studies use the terms exoelectrogens [2] and electricigens [3]) is necessary for further improvement and reliable operation of MFCs. In order to identify what organisms are involved in electricity generation, microbial communities established in anode chambers have been analyzed by molecular ecological approaches [7-9]. Although metal-reducing bacteria, such as *Geobacter *[10] and *Shewanella *[11], have been considered as model EGMs, they have not always been detected in MFCs, and electrogenic populations seem to be more diverse than previously thought [7-9]. Physiological studies aim to reveal how these microorganisms transfer electrons to anodes. Recent studies have suggested that *Geobacter *and *Shewanella *use electrically conductive extracellular filaments (termed nanowires) for transferring electrons to solid electron acceptors, such as graphite anodes [12-14]. However, it is unclear whether or not other EGMs occurring in MFCs also use nanowires. Many important questions remain unanswered concerning microbiology of electricity generation (electrogenesis).

In this study, electrogenic communities established in a cellulose-fed MFC were analyzed. Cellulose was selected as a substrate, because it is one of the major constituents of waste biomass. Previous studies have analyzed cellulolytic methanogenic communities in anaerobic digesters [15,16]; but so far only a few studies have examined cellulolytic electrogenic communities [17]. Our analysis here emphasizes the morphological characterization and phylogenetic identification of microorganisms in biofilms established on graphite anodes, which allowed us to suggest a bacterial lineage important for electricity generation.

## Results

### Electricity generation from cellulose

An H-type two-chamber MFC similar to those previously reported [1,10,18] was constructed, and its performance was evaluated using *Geobacter sulfurreducens *[10] as a model EGM. It was demonstrated that the reactor performance was equivalent to those previously [10].

**Figure 1:**
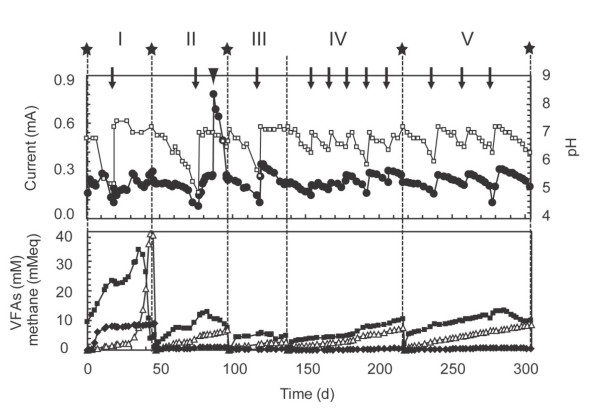
**Current generation and metabolite concentrations in the cellulose-fed two-chambered MFC**. Closed circle, electric current (mA); open square, pH in the anode chamber; closed square, acetate in the anode chamber (mM); closed diamond, propionate in the anode chamber (mM); open triangle, methane in the anode chamber. Methane concentration was expressed as 'mM equivalent (eq.)' by supposing that all methane was present in the aqueous phase. Broken lines represent times when the anode electrode was transferred to new anode chambers, solid stars indicate times when cellulose (6 g l^-1^) was added to the anode chambers, while arrows indicate times when pH in the anode chamber was adjusted to 7.0. The arrowhead indicates the time when the cathode chamber was supplemented with potassium ferricyanide.

The anode chamber of the MFC was inoculated with the rice paddy field soil and supplemented with cellulose. Current outputs of approximately 0.2 mA were immediately detected (Fig. 1), while no current was observed in the control MFC (no soil inoculation) (data not shown). During the initial 25 days, acid fermentation proceeded in the anode chamber, resulting in the accumulation of acetate and propionate and a drop in the current. After the pH was adjusted to 7 by adding a Na_2_CO_3 _solution, methane was actively produced (from day 30 to 45 in phase I).

When the cation-exchange membrane cracked and the anode medium leaked into the cathode chamber due to a high gas pressure in the anode chamber, the biofilm-harbouring anode was transferred to a new anode chamber containing fresh medium. In these phases of subcultures (phases II to V), the MFC system also produced a current of 0.2 mA to 0.3 mA (the maximum current of 0.31 mA, equivalent to 10 mW m^-2 ^[per anode surface] and 0.15 W m^-3 ^[per anode-chamber working volume]), while methane production was slower than that in phase I. Hydrogen was not detected (below 5 Pa in the headspace). Production rates of electron (estimated from electric currents), methane, hydrogen and volatile fatty acids (VFAs) in these phases are summarized in Table 1. Acetate was produced and accumulated constantly, while only small amounts of the other VFAs were produced. Short-chain alcohols (ethanol, propanol and butanol) were not detected throughout the experiment. The pH in the anode chamber gradually dropped and was occasionally adjusted to 7 by adding a Na_2_CO_3 _solution (Fig. 1). Electric current was tightly linked to pH fluctuations. As shown in Table 1, the performance of the MFC became stable in phases IV and V, indicating that the enrichment of microbial populations was accomplished in these phases. When potassium ferricyanide was added to the cathode chamber (from days 87 to 96 in Fig. 1), the electric current was increased to approximately 0.8 mA. The methane-production rate decreased from 0.44 mmol day^-1 ^to 0.32 mmol day^-1 ^during this period (Table 1). The coulombic efficiency was not calculated, since the rate of cellulose degradation could not be accurately determined.

Resazurin and cysteine may act as electron shuttles and substrates, respectively. When resazurin was omitted from the anode medium, the same level of current was generated (data not shown), suggesting that other electron-transfer mechanisms existed in our MFC. The amount of cysteine added was less than one tenth than that of cellulose. Considering the results of a previous study [18] where cysteine was shown to be the sole carbon source for electricity generation, it is likely that cysteine was rapidly depleted and did not largely affect methane and electricity production in latter days of each phase. In fact, cysteine was depleted within 14 days after commencing a new phase of enrichment culture (data not shown).

**Table 1:** Metabolite-production rates in the cellulose-fed MFC^a^

Enrichment phase	Day^b^	Metabolite-production rate (mmol l^-1 ^d^-1^)					
		
		Electron	Methane	Acetate	Propionate	Butyrate	Hydrogen
I	0 to 32	0.59	0.10	0.51	0.25	0.05	0.00
I	35 to 45	0.64	3.66	-3.60^c^	0.02	0.03	0.00
II	46 to 82	0.54	0.15	0.32	0.02	0.00	0.00
II^d^	87 to 96	1.55	0.11	-0.31^c^	0.00	0.00	0.00
III	97 to 136	0.65	0.06	0.08	0.00	0.00	0.00
IV	140 to 210	0.65	0.08	0.09	0.01	0.00	0.00
V	220 to 273	0.69	0.09	0.12	0.01	0.00	0.00

^a ^Short-chain alcohols (ethanol, propanol and butanol) were not detected throughout the experiment.

^b ^Data obtained in these days were used for calculation.

^c ^Acetate was consumed.

^d ^Ferricyanide was added to the cathode chamber.

### Biofilm morphology

In phases II to V, most of the microorganisms adhered onto the anodes and formed biofilms. The culture media in the anode chamber became a transparent brown color, and cell concentrations were below 10^7 ^cells ml^-1 ^(as determined by the DAPI [4',6-diamino-2-phenylindole] direct count method using an epifluorescence microscope [19]). In order to examine the morphology of anode biofilms, biofilm samples were taken on day 216 (phase IV) and subjected to field emission-scanning electron microscopy (FE-SEM).

**Figure 2:**
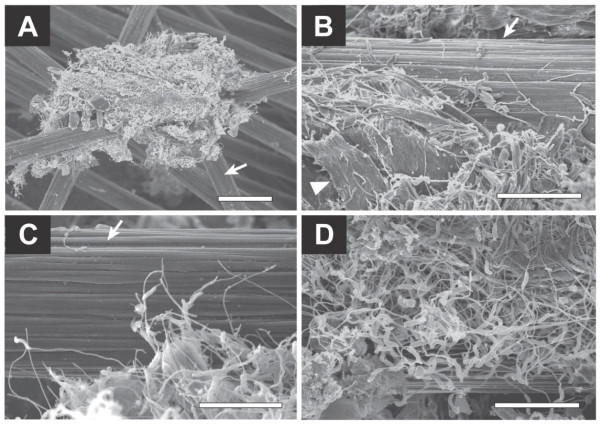
**FE-SEM images for anode biofilms adhering onto graphite-fiber anodes (day 216)**. Arrows indicate graphite fibers, while an arrowhead indicates a cellulose fiber. The bar in panel A is 20 μm, while those in the other panels are 5 μm.

**Figure 3:**
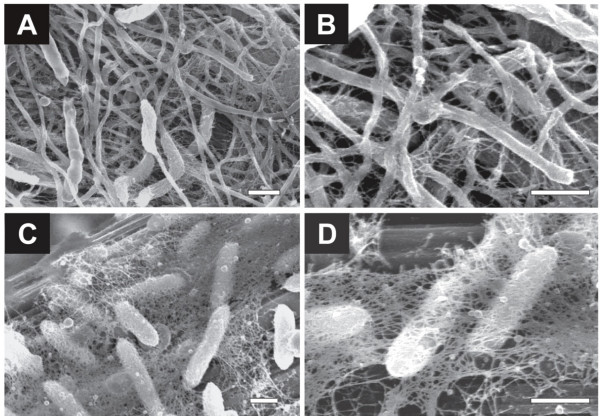
**Magnified FE-SEM images for biofilms adhering onto graphite-fiber anodes**. Anode biofilms enriched from the rice paddy field soil were shown in panels A and B, while *Geobacter *biofilms were shown in panels C and D. Bars are 500 nm.

Electron micrographs revealed unique biofilm structures and cell shapes (Figs. 2 and 3). Photos show that biofilms were sparsely distributed on the graphite fibers (Fig. 3A) along with highly complicated structures comprised of morphologically different cells and cellulose fibers (Fig. 2B). Many rod-shaped cells had long filamentous appendages (approximately 100 nm in thickness) that, as seen at the edge of the biofilm (Figs. 2C), bridged between microbial cells and the electrodes. In other cases, cells themselves were filamentous and occasionally intertwined with each other (Fig. 2D). Rods with filamentous appendages seemed to be more abundant than filamentous cells.

Magnified electron micrographs revealed the presence of large amounts of thin, thread-like appendages (approximately 10 nm in thickness) that interconnected microbial cells and thick filamentous appendages (Fig. 3AB). We also observed *G. sulfurreducens *PCA cells adhering onto the anode electrodes (Fig. 3CD) and found that they had an abundance of thin network filaments (20 to 30 nm in thickness) that were somewhat thicker than the thin thread-like appendages in the soil-derived biofilms. These observations revealed that a variety of filamentous structures were present in the anode biofilms.

### Phylogenetic composition

The unique morphology of the anode biofilm prompted us to identify what organisms constituted these biofilms. For this purpose, we constructed 16S rRNA gene clone libraries for the phases I to V communities, and compared them to each other. As described in previous papers, 16S rRNA gene clone library analyses suffer from the biases associated with PCR amplification [20]. To obviate this limitation as much as possible, we selected the universal PCR primer set that has been reliably used in previous studies (for example, reference [21]), and PCR cycles were minimized. In addition, we constructed clone libraries for a microbial community in the soil and those in the MFC in the different phases and compared phylotypes in these libraries, allowing us to identify sequences that specifically occurred in one library.

**Figure 4:**
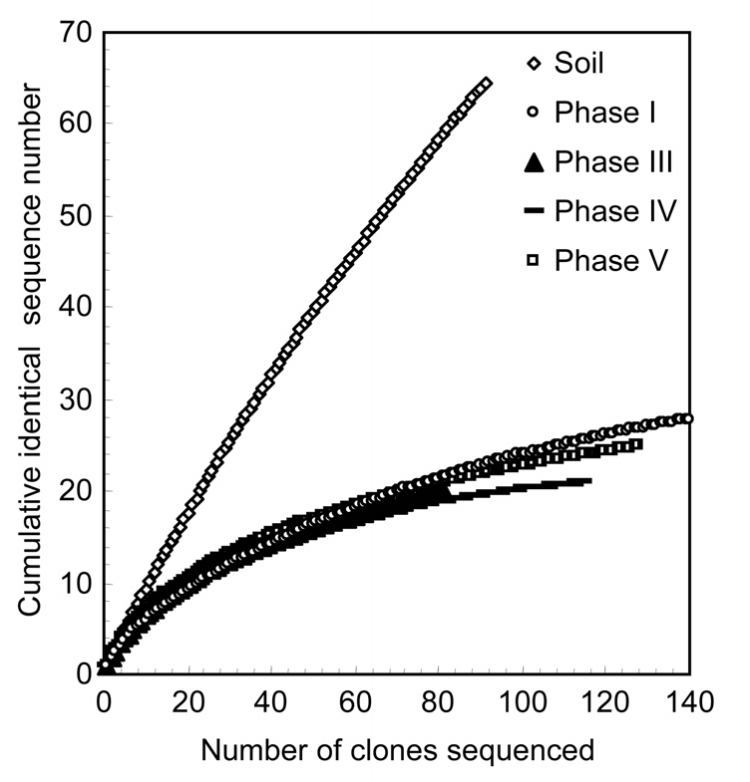
Rarefaction curves for the different phylotypes obtained from the 16S rRNA gene clone libraries for the original rice paddy field soil and the anode biofilms.

The rarefaction-curve analysis (Fig. 4) showed that the biofilm communities were much less diverse than the soil community; the biofilm libraries seemed to be comprised of 20 to 30 different phylotypes. This analysis indicates that the biofilm libraries covered most organisms in the anode biofilms.

Results of the sequence analysis are summarized in Table 2 and show the phylogenetic affiliations of the major phylotypes (those including more than 5 clones). Information regarding the minor phylotypes (indicated as others in Table 2) is provided upon request to the authors. It was shown that most clones were affiliated with the domain *Bacteria*, while others were affiliated with the *Archaea *(closely related to *Methanobacterium*). No sequences of the *Eukarya *were obtained. Large portions (80% to 90%) of the biofilm clones were categorized into the major phylotypes (Table 2). The libraries for biofilms in the functionally stable phases (phases IV and V) were comprised of 7 or 8 major phylotypes (those including more than 5 clones); among them, 2 *Clostridiales *(Mfc-2 and Mfc-3), *Chloroflexi *(Mfc-6), *Rhizobiales *(Mfc-9) and *Euryarchaeota *(Mfc-11) were stably present in these libraries. Mfc-5 increased in the phase-V library, while Mfc-8 decreased. The stably present phylotypes may have represented important populations in these phases of MFC.

**Table 2:** Major phylotypes obtained from the cellulose-fed MFC^a^

Major phylotype	No. of clone in each library^b^					Database match (Accesion no.)
		
	Soil (day 0)	Phase I (day 46)	Phase III (day 137)	Phase IV (day 216)	Phase V (day 306)	
***Firmicutes***						
Mfc-1	0	32	25	5	4	95% *Ethanoligenens harbinense *YUAN-3 (AY295777)
Mfc-2	0	3	5	9	7	95% *Clostridium *sp. Z6 (AY949859)
Mfc-3	0	0	5	9	11	94% *Propionispora hapie *(AJ508928)
Mfc-4	0	43	0	0	0	88% *Clostridium *sp. JC3 (AB093546)
Mfc-5	0	0	0	2	22	99% *Clostridium celerecrescens *(X71848)
Others^c^	5	7	4	6	7	
***Chloroflexi***						
Mfc-6	0	3	6	19	22	92% *Leptolinea tardivitalis*YMTK-2 (AB109438)
Others^c^	5	0	0	0	0	
***Bacteroidetes***						
Mfc-7	0	22	5	5	3	91% *Paludibacter propionicigens *(AB078842)
Others^c^	6	7	1	0	0	
***Spirochaetes***						
Mfc-8	0	6	4	21	6	92% *Treponema *denticola ATCC35405 (AE017226)
Others^c^	0	4	0	0	0	
***Proteobacteria***						
Mfc-9	0	0	1	23	22	97% *Rhizobiales *bacterium RR54 (AB174822)
Mfc-10	0	3	7	1	3	96% *Myxobacterium *sp. KC (AF482687)
Soil-1	9	0	0	0	0	99% *Achromobacter xylosoxidans *(AF411021)
Soil-2	7	0	0	0	0	99% *Stenotrophomonas maltophilia *E2 (AY841799)
Others^c^	26	0	1	5	2	
***Euryarchaeota***						
Mfc-11	0	2	8	10	9	99% *Methanobacterium bryantii *RiH2 (AF028688)
Others^c^	0	1	2	0	2	
**Other phyla and unknown clones**						
Others^c^	34	9	1	0	5	

Total clone	93	142	75	115	129	

Ratio (%)^d^	17	80	88	90	85	

^a ^The Major phylotype was defined as a phylotype containing more than 5 clones.

^b ^Names for the libraries correspond to the subculture phases of MFC.

^c ^For a list of other phylotypes (minor phylotypes), refer to the supplementary materials that may be found in the website (Table S1).

^d ^Ratio in number of clones affiliated with the major phylotypes to the total clones.

### Fluorescence in-situ hybridization (FISH)

**Figure 5:**
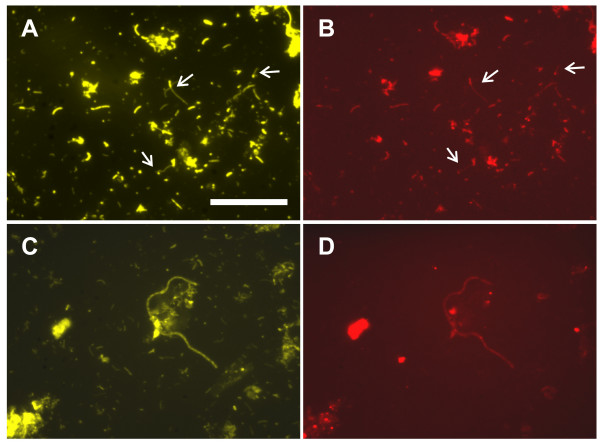
**FISH images for organisms represented by phylotypes Mfc-6 (A, B) and Mfc-9 (C, D)**. Images for Nano orange-stained (A, C) and FISH probes-labeled (B, D) cells are presented. The scale bar (50 μm) in panel A applies to all panels. FISH-labeled rods with filamentous appendages are indicated with arrows.

Among the stably present phylotypes, Mfc-6 is affiliated with *Chloroflexi *Subphylum I that is comprised of filamentous microorganisms present in anaerobic digesters [22,23], whereas Mfc-9 is affiliated with *Rhizobiales *that include prosthecate bacteria (e.g, *Hyphomicrobium *[24]) known to possess filamentous appendages called 'prosthecae' [25-27]. In order to investigate the cell morphologies of bacteria represented by these phylotypes, we carried out FISH with specific oligonucleotide probes Hypho1241 [24] and GNSB941 [22]. The nucleotide sequence of probe Hypho1241 was 100% matched with Mfc-6, and the other phylotypes obtained in the present study had more than two mismatches. GNSB941 was 100% matched with phylotypes affiliated with *Chloroflexi *Subphylum I, among which Mfc-6 was the only phylotype abundantly obtained; non-*Chloroflexi *Subphylum I phylotypes had more than two mismatches. As shown in panel D in Fig. 5, the FISH result shows that GNSB941 labelled filamentous cells. On the other hand, Hypho1241 labelled rod-shaped cells (panel B), and Nano orange staining showed that some of these cells had filamentous appendages (panel A). Cells with filamentous appendages were relatively few, probably because filamentous cells were difficult to be detached from the biofilm. Nevertheless, it was shown that Hypho1241-labeled cells were abundantly present (38% ± 5% [SD, n = 5] of the Nano orange-stained total cells).

## Discussion

The present study investigated the electrogenic biofilm communities established in the anode chamber of a cellulose-fed MFC. In our MFC system, methane was produced along with current (Fig. 1 and Table 1). As 8 moles of electrons are used to biologically produce one mole of methane from carbon dioxide, it is estimated that methane production and electricity generation nearly equally contributed to the terminal electron-accepting reaction in the stable enrichments (phases IV and V). When the cathode was supplemented with ferricyanide (day 87), however, the current was increased to 0.8 mA, while the methane-production rate decreased. In addition, when the external resistor was changed to 100 Ω, the current was increased to over 0.5 mA (data not shown). These results indicate that the electricity generation was suppressed by instrumental limitations of the MFC system. We therefore conclude that electricity generation is superior to methane production in the terminal electron-accepting reaction coupled to cellulose oxidation. From the metabolite analysis, acetate was considered to be one of the major intermediate metabolites of cellulose degradation and possibly used to generate electricity.

The FE-SEM observation revealed three distinctive morphological features of biofilm organisms in the cellulose-fed MFC (Figs. 2 and 3). First, many cells were straight filaments, while some were spiral. Second, many rod-shaped cells had thick filamentous appendages. Third, filamentous cells and thick filamentous appendages were interconnected to each other via thin filaments. Several previous studies have reported SEM pictures of biofilms on anodes [7,10], while no reports have documented the presence of filamentous cell structures in these biofilms. Several factors can be considered to explain this difference. First, there were differences in the MFC operational conditions, including organic substrates, inorganic ingredients and seed inocula. In addition, our high-resolution FE-SEM may have facilitated the detection of thin filaments of the biofilm microorganisms, including the network filaments of *Geobacter *(Fig. 3). Recent studies have suggested that metal-reducing bacteria, such as *Geobacter *and *Shewanella *species, utilize electrically conductive nanowires to discharge electrons to extracellular electron acceptors [12-14]. Interestingly, we found that the naturally occurring electrogenic biofilm (enriched from soil) produced an abundance of filaments that connected the microbial cells to the electrode and interconnected to each other. There have been several reports presenting microscopic observations of microbial communities engaged in methanogenic cellulose degradation [15,16]; however, filamentous structures were not abundant in these communities. In addition, methanogenic biofilms enriched on graphite fibers from the same soil did not include filamentous appendages (our unpublished data). From these data, we postulate that the filamentous structures should be important for electron transfer to the anode, although other mechanisms, such as the use of electron shuttles [9], should also be taken into account.

Environmental rRNA gene sequences have been used to infer properties and ecological roles of organisms that they represent [21,28], although this type of analysis may only be possible if some common properties are known for isolated organisms affiliated with the same phylogenetic group. This study attempted to deduce ecological roles of microorganisms represented by the major phylotypes in the stable enrichment phases (phases IV and V in Table 2). Two major phylotypes, Mfc-2 and Mfc-3, were affiliated with the order *Clostridiales *and closely related to the genera *Clostridium *[29] and *Propionispora *[30], respectively. These genera have been known to include anaerobic fermentative organisms that degrade complex (e.g., cellulose) and simple (e.g., glucose) carbohydrates. Since the other major phylotypes were not related to cellulolytic anaerobes, we deduce that these clostridia were involved in cellulose degradation. Phylotype Mfc-6 was affiliated with *Chloroflexi *subphylum I [22,23]. Organisms affiliated with this subphylum are known to be filamentous organisms and ferment sugars [23]. Cell morphology of this group of organisms in the MFC was confirmed by the FISH analysis (Fig. 5). Mfc-7 and Mfc-8 were affiliated with the orders *Bacteroidales *and *Spirochaetales*, respectively. Organisms in these groups have commonly been detected in anaerobic digesters (for example, reference [31]). Although the ecological roles of these organisms in the anaerobic digestion are unclear, they are considered to ferment small carbohydrates and amino acids [31]. In addition, Mfc-11 should have represented hydrogenotrophic methanogens.

Mfc-9 was affiliated with the order *Rhizobiales*. The database search indicated that it was closely related to strain RR54, a bacterium isolated from the roots of rice plants (an unpublished strain only found in the nucleotide sequence databases). In addition, Mfc-9 was also related to strains belonging to *Rhizobiaceae *and *Hyphomicrobiaceae*. A common feature of these organisms is that they are facultatively aerobic heterotrophs [32], suggesting that they have respiratory electron-transport chains. Another feature shared by many strains in this group is that they possess one or several long cylindrical appendages called 'prosthecae' [25-27]. Prosthecae are sticky filaments that play important roles when prosthecate bacteria adhere onto solid surfaces [25]. The prosthecae-like appendages of Mfc-9 bacteria were shown in the FISH analysis (Fig. 5). The FISH data also confirmed that Mfc-9 bacteria were abundantly present in the anode biofilms. It is also noteworthy that bacteria belonging to the *Rhizobiaceae *are known to have efficient uptake hydrogenases that allow them to recycle hydrogen generated in the nitrogen fixation process within legume nodules [33,34]. Most importantly, different from the other major phylotypes obtained in the present study, Rhizobiales sequences have not been found in cellulolytic methanogenic communities in anaerobic digesters [15,16]. We have conducted the phylogenetic analysis of a biofilm community attaching onto graphite fibers that was enriched from the same paddy field soil and engaged only in methanogenic cellulose degradation; in that analysis, no *Rhizobiales *phylotype was recovered (our unpublished data). From these analyses, we suggest that Mfc-9 abundantly occurred in the anode biofilm in response to electricity generation and may have been involved in the electricity generation. In order to investigate their activities and physiology, they should be isolated for pure-culture studies. In addition, we are also interested in investigating properties (e.g., electric conductivity and adhesiveness) and the roles of thick and thin filaments abundantly present in the anode biofilm.

## Conclusion

In a MFC reactor, a microbial community enriched from rice paddy soil generated electricity of up to 0.3 mA by utilizing cellulose as the energy source. Microbiological analyses revealed that *Rhizobiales *bacteria with filamentous appendages constituted the major population in the anode biofilm. Comparative analyses based on available physiological information of closely related bacterial isolates suggest that they were possibly involved in the electricity generation. Isolation of these *Rhizobiales *bacteria will deepen our understanding of how microbes generate electricity from cellulose.

## Methods

### Microorganisms and culture conditions

*G. sulfurreducens *PCA (= DSM 12127) was obtained from Deutsche Sammlung von Mikroorganismen und Zellkulturen GmbH. The growth medium contained the following (per liter): 0.1 g of KCl, 0.2 g of NH_4_Cl, 0.6 g of NaH_2_PO_4_, 10 ml of a vitamin solution [35], 10 ml of a trace element solution [35], 50 μl of a titanium citrate solution [36], 10 mM acetate and 40 mM fumarate. Cultivation was conducted in a bottle (125 ml in capacity, sealed with a Teflon-coated butyl rubber septum and secured with a crimped aluminum cap) containing 50 ml of the medium at 30°C under an atmosphere of N_2 _and CO_2 _(80/20 [v/v]) with shaking. Cultivation was initiated by inoculating with 5 ml of a preculture in the same medium. Rice paddy field soil was obtained at Kamaishi, Japan in August 2005.

### MFC configuration and operation

An H-type two-chamber MFC similar to those previously reported [1,10,17] was constructed using two glass vessels (each 450 ml in capacity) connected with a glass tubing and a pinch-clump assembly. Liquids in the two chambers were separated by a cation-exchange membrane (Neosepta CIMS, Astom). The top of each chamber was covered with a glass dome with three sampling ports. All junctions and sampling ports were sealed with tight butyl-rubber stoppers. Anode electrodes were bundled graphite fibers (6 μm in diameter, 5000 fibers per anode) (Sogo carbon, Yokohama), while a cathode electrode was an unpolished graphite plate with no catalyst (3 cm × 10 cm × 0.5 cm) (the Fuel cell store Japan, Hamamatsu).

After sterilization, the anode chamber was filled with 300 ml of a sterilized anaerobic MFC medium comprised of 0.1 g of KCl, 0.2 g of NH_4_Cl, 0.6 g of NaH_2_PO_4_, 1 ml of the vitamin solution, 1 ml of the trace element solution, 1 ml of a Se/W solution [35], 1 mg of resazurin and 0.5 g of L-cysteine per liter (pH 6.8) and supplemented with 6 g l^-1 ^cellulose (Avicel, Asahi Kasei) as a substrate. The headspace of the anode chamber was filled with N_2 _and CO_2 _(80/20 [v/v]). The cathode chamber was filled with 300 ml of a sterilized 30 mM Tris-HCl buffer (pH 6.8) supplemented with 0.1 g of KCl, 0.2 g of NH_4_Cl and 0.6 g of NaH_2_PO_4 _and bubbled with the filter-sterilized air. Potassium ferricyanide (20 mM) was occasionally used as an oxidizing agent in the cathode chamber. The anode chamber was inoculated with rice paddy field soil (3.5 g [wet weight]) and slowly agitated using a magnetic stirrer. The MFC reactor was operated in a temperature-controlled room (at 30°C). The two electrodes were connected with an electric cable and an external resistor (510 Ω), and a voltage across the resistor was measured using a potentiostat (multipotentiostat 2092, Toho Giken). An electric current was converted to moles of electron using the following equations and constants; 1 C = 1 A × 1 s, 1 C = 6.24 × 10^18 ^electrons, and 1 mol = 6.02 × 10^23 ^electrons (96,500 C mol^-1^). The pH of the anode medium was occasionally adjusted to 7 by adding a Na_2_CO_3 _solution (5% [w/v]). When the cation-exchange membrane was cracked or the electric current dropped independent of pH, the biofilm-bearing anode electrodes were taken out from the anode chamber and transferred to a new anode chamber containing the fresh medium; this was done in an anaerobic glove box filled with N_2 _and CO_2 _(80/20 [v/v]).

### Chemical analyses

After a liquid sample was passed through a 0.22 μm pore membrane (type GV, Millipore), volatile fatty acids were analyzed using a high-pressure liquid chromatography (Organic acid analysis system, Shimadzu) equipped with CDD detector and dual packed columns (Shim-pack SCR102-H, Shimadzu). As an eluant, a mixture of an equal volume of 5 mM *p*-toluenesulfonic acid solution and 20 mM Bis-Tris solution containing 5 mM *p*-toluenesulfonic acid and 100 μM EDTA was used at 0.8 ml min^-1^. The filtrate was acidified with concentrated HCl, and short-chain alcohols were analyzed using a gas chromatograph (GC-2010; Shimadzu) with a flame ionization detector and a DB-FFAP column (Shimadzu). Methane, hydrogen, nitrogen and carbon dioxide at the headspace of the anode chamber were measured using a gas chromatograph (GC-14A, Shimadzu) equipped with a thermal conductivity detector and parallel packed columns (molecular sieve 5A 60–80/porapack Q 80–100, Shimadzu) as described previously [35]. Cysteine was measured as described elsewhere [36].

### FE-SEM

FE-SEM was performed as described previously [35]. A small portion of graphite fibers harbouring biofilms were carefully removed from the anode electrode, and the biofilm cells were fixed with 1.25% glutaraldehyde and 1.3% osmium tetraoxide. After cells were dehydrated using a graded series of ethanol solutions, they were dried using an HCP-2 drier (Hitachi). The resultant specimen was coated with osmium using a CVD coating device (Hitachi) and observed under an S4500 FE-SEM (Hitachi) at 5 kV.

### PCR amplification, cloning and sequencing of 16S rRNA gene fragments

Total DNA was extracted from the rice paddy field soil (0.5 g) and biofilms on graphite fiber electrodes (approximately 0.5 g) using FAST DNA spin kit for soil (Q-BIO gene) according to the manufacturer's instruction. PCR amplification of 16S rRNA gene fragments was performed using U515f (5'-CTGYCAGCMGCCGCGGTAA-3', nucleotide position 515 to 533 in the Escherichia coli sequence) as a forward primer and U1492r (5'-GGYTACCTTGTTACGACTT-3', nucleotide position 1492 to 1510) as a reverse primer. A PCR solution (50 μl) contained 1.25 U of Taq DNA polymerase (Amplitaq Gold, Applied Biosystems), 10 mM Tris-HCl (pH 8.3), 50 mM KCl, 1.5 mM MgCl_2_, 0.001% (w/v) gelatin, each deoxynucleoside triphosphate at a concentration of 200 μM, 50 pmol of each primer and an appropriate amount of template DNA. The amplification conditions were as follows: an initial step of 94°C for 10 min; 25 to 30 cycles consisting of 94°C for 1 min, 50°C for 1 min and 72°C for 2 min; a final elongation step at 72°C for 10 min. The PCR cycles were set at minimum values at which sufficient quantities of products were obtained. Amplified fragments were purified with a QIAquick PCR purification kit (QIAGEN), ligated into the pGEM-T vector (Promega) and cloned into *Escherichia coli *competent cells as described previously [19]. Vector harbouring clones were selected on Luria-Bertani plates supplemented with ampicillin (50 μg ml^-1^). PCR-amplified 16S rRNA gene fragments were recovered from colonies by PCR using primers T7W (5'-TAATACGACTCACTATAGGGC-3') and SP6W (5'-ATTTAGGTGACACTATAGAATACTC-3') (the primers targeted the pGEM-T vector sequences flanking the insertion) as described previously [19]. Clones containing appropriate sizes of the insertion were selected by the electrophoresis analysis, and their nucleotide sequences were determined as described previously [19]. A rarefaction analysis was conducted using the Analytic Rarefaction program [37].

### Phylogenetic analyses

Sequences of 16S rRNA genes determined in this study were aligned to each other using Clustal W version 1.7 [38] and assigned to phylotypes (classified as a unique clone or group of clones with sequence similarity of >0.98). Database searches for related 16S rRNA gene sequences were conducted using the BLAST program [39] and the GenBank nucleotide sequence database. Checks for chimeric sequences were conducted using the chimera check program in the Ribosomal Database Project database [40].

### FISH

Probes Hypho1241 (targeting *Hyphomicrobium *and related *Rhizobiales *bacteria [24]) and GNSB941 (targeting Chloroflexi Subphylum I bacteria [22]) labelled with Cy5 were used. Hybridization conditions (see below) were determined by theoretical analyses [41] and in vitro hybridization experiments [42] using positive and control 16S rRNA sequences cloned in the present study; nucleotide sequences with more than two mismatches were not hybridized with these probes under conditions described below. Bacterial cells attaching onto anode fibers were detached by vortexing in phosphate-buffered saline [19], and it was checked by microscopic observation. Cells were fixed in a 4% (wt/vol) paraformaldehyde solution for 4 h at 4°C. The cells were attached to APS-coated glass slides (Matsunami) and dehydrated by sequential washes in 50, 75, and 98% (vol/vol) ethanol (2 min each). Subsequently, 20 μl of hybridization solution (0.9 M NaCl, 20 mM Tris-HCl [pH 7.2], 0.01% [wt/vol] sodium dodecyl sulfate, 10% [wt/vol] formamide) containing 100 ng of a probe was added to each hybridization well and was incubated at 45°C for overnight under a humid condition. Slides were washed twice in the hybridization solution at 48°C for 5 min before cells were stained with Nano orange (Molecular probes). Nano orange (a protein-binding fluorescent dye) was used for staining total microbial cells. The Cells were overlaid with an anti-fading reagent (VectaShield, Vector) and observed under a fluorescence microscope (BX60, Olympus) equipped with a CCD camera (PXL1400, Photometrics). Images were analyzed using the Photoshop software (Adobe), and numbers of probe-labeled cells and those of Nano orange-stained cells were counted on a computer screen. More than 100 Nano orange-stained cells in five sights were counted to obtain a ratio.

### Accession numbers

The nucleotide sequences reported in this paper have been deposited in the GSDB, DDBJ, EMBL and NCBI nucleotide sequence databases under accession nos. AB286216 to AB286330.

## Authors' contributions

SI performed most of experiments and wrote the manuscript. TS performed FISH, and YH performed the FE-SEM analysis. KW designed the work and wrote the manuscript.
